# Use of Folk Therapy in Taiwan: A Nationwide Cross-Sectional Survey of Prevalence and Associated Factors

**DOI:** 10.1155/2015/649265

**Published:** 2015-06-11

**Authors:** Chun-Chuan Shih, Lu-Hsiang Huang, Hsin-Long Lane, Chin-Chuan Tsai, Jaung-Geng Lin, Ta-Liang Chen, Chun-Chieh Yeh, Chien-Chang Liao

**Affiliations:** ^1^School of Chinese Medicine for Post-Baccalaureate, I-Shou University, Kaohsiung 82445, Taiwan; ^2^Taipei Chinese Medical Association, Taipei 100, Taiwan; ^3^Quality of Traditional Chinese Medicine and Pharmacy Medical Association, New Taipei City 247, Taiwan; ^4^Ph.D. Program for Clinical Drug Discovery from Botanical Herbs, Taipei Medical University, Taipei 110, Taiwan; ^5^School of Chinese Medicine, College of Chinese Medicine, China Medical University, Taichung 40402, Taiwan; ^6^Clinical Informatics and Medical Statistics Research Center, Chang Gung University, Taoyuan 33302, Taiwan; ^7^Department of Anesthesiology, Taipei Medical University Hospital, Taipei 110, Taiwan; ^8^Health Policy Research Center, Taipei Medical University Hospital, Taipei 110, Taiwan; ^9^School of Medicine, Taipei Medical University, Taipei 110, Taiwan; ^10^Department of Surgery, China Medical University Hospital, Taichung 40402, Taiwan; ^11^Department of Surgery, University of Illinois, Chicago, IL 60612, USA

## Abstract

*Background*. This study investigates the prevalence of and factors associated with users of folk therapy in Taiwan. *Methods*. Using data from the 2005 National Health Interview Survey and the National Health Insurance Research Database, we identified 16,750 adults aged 20 years and older. Sociodemographic factors, lifestyle, medical utilization, and health behaviors were compared between people using and not using folk therapy. Adjusted odds ratios (ORs) and 95% confidence intervals (CIs) of factors associated with folk therapy were analyzed. *Results*. The one-month prevalence of folk therapy use was 6.8%, which was significantly associated with ages of 30–59 years (OR = 1.98, 95% CI = 1.49–2.63), women (OR = 1.63, 95% CI = 1.40–1.90), nonindigenous population (OR = 1.90, 95% CI = 1.14–3.17), having two or more unhealthy lifestyle habits (OR = 1.51, 95% CI = 1.26–1.81), high density of traditional Chinese medicine (TCM) physicians (OR = 1.40, 95% CI = 1.20–1.62), and being ill without receiving medical care in past six months (OR = 2.11, 95% CI = 1.76–2.53). Medical care utilization of TCM and Western medicine were also associated factors for folk therapy. *Conclusions*. The use of folk therapy is correlated with sociodemographics, lifestyle and health behaviors.

## 1. Introduction

The acceptance and use of complementary alternative medicine (CAM) in general populations has been increasing in both Asian and Western countries [1–5]. A population-based survey in the United Kingdom showed that use of one or more CAM therapies during the life course was 46% [3]. A high prevalence of CAM use has also been noted in the United States among patients with breast cancer [5]. People using CAM may intend to improve their health and well-being [6, 7], to relieve symptoms associated with chronic or terminal illness, or to attenuate side effects of Western medical treatments [8, 9]. Different CAM types address different issues; these include physical, biochemical, nutritional, emotional, social, and spiritual aspects of illness and wellness.

Eisenberg suggests that physicians need to understand patients' use of unconventional therapy whenever they obtain medical histories [10]. Traditional Chinese medicine (TCM) is commonly used in Taiwan [11–15] and many other Asian countries [11–15]. While Western medicine enjoys credibility worldwide, different forms of CAM are also well established and commonly used in India, China, Korea, Taiwan, and other countries [11–19]. Estimated one-month, one-year, and six-year utilization of TCM service in Taiwan prior to this survey were 10.4%, 28.4%, and 62.5%, respectively [16, 17]. Previous studies indicated that users of CAM tend to be female, highly educated, with poor health status, or lacking access to conventional medicine [20–24].

Tsai et al. [25] reported that 1.3% of adult Taiwanese use folk therapies. These are defined as various types of TCM delivered by nonlicensed practitioners and non-board-certified specialists who are not reimbursed by Taiwan's National Health Insurance Program, so these patients pay 100% out of pocket for such treatments. As many people use folk therapy delivered by NHS-reimbursed practitioners and because the use of folk therapy is still not completely understood in Taiwan, we conducted a cross-sectional study by using data from the 2005 National Health Interview Survey (NHIS) and the National Health Insurance Database to investigate the prevalence of folk therapy use and its associated factors in Taiwan.

## 2. Materials and Methods

### 2.1. Study Design

In 2005, the National Health Research Institute and the Bureau of Health Promotion conducted the NHIS using face-to-face questionnaire interviews [26, 27]. The population of Taiwan is approximately 23 million people who live in 7 cities and 18 counties. The NHIS included a representative sample of 24,726 interviews with noninstitutionalized population conducted from January to December 2005. Interviewees were 15 years and older, and each interview was performed at the subject's home. These subjects were selected from the household census.

Using a standardized face-to-face questionnaire interview, the NHIS used a multistage, stratified systematic sampling scheme to collect a nationally representative sample of the nation's population. The response rate was 80.6% for individual subjects. At the survey's end, participants were asked for permission to access their National Health Insurance records for research purposes. All study participants signed the informed consent to link their information with the National Health Insurance claims data to retrieve information on their use of medical services in 2005. This study was evaluated and approved by Taiwan's National Health Research Institutes. This study analyzed 16,750 study participants aged 20 years and older.

### 2.2. Data Collection

The current study used both NHIS data and 2005 National Health Insurance claims data. With the written informed consent of eligible NHIS participants, the 2005 NHIS data were linked to the 2005 National Health Insurance claims data. Information about prevalence and frequency of TCM and Western medicine use was drawn from the claims data. The survey data provided information on use of folk therapy as well as sociodemographic data such as age, gender, education, occupation, family income, ethnicity, religion, marital status, and whether respondents had regular health checkups or unhealthy lifestyle habits including smoking, alcohol use, and betel nut chewing (betel nut users chew a combination of betel leaf, areca nut, and slaked lime; this is a habitual practice in many parts of Asia but is also identified as an important risk factor for oral cancer) [28, 29].

### 2.3. Definitions and Measures

Folk therapy use is defined as utilization of any of the following within the month before the interview: gua-sha (skin scraping), tui-na (massage and kneading), ba-guan (cupping or vacuum bottle therapy), bone setting, spine alignment, qigong, divination, written charms, shaman consultation, talismans, incense ash, and other related practices. The difference between TCM and folk therapy lies in their respective legal statuses. At least four differences exist between TCM and folk therapy in Taiwan. First, folk therapy practitioners provide services in nonclinical settings or facilities, while TCM physicians perform medical services at clinics or hospitals. Second, folk therapy practitioners lack licenses and recognized clinical training, while TCM physicians have government-issued licenses and take hospital-based clinical training [30]. Third, folk therapy is not covered by the National Health Insurance Program, while TCM has been covered since 1995. Fourth, folk therapy practitioners have little scientific evidence that their services have therapeutic effects, yet TCM physicians perform medical services that have shown therapeutic effects that are widely recognized in professional literature.

The questions of medical utilization in the questionnaire are as follows. In the past one month, have you undergone any therapy such as gua-sha (skin scraping), tui-na (massage and kneading), ba-guan (vacuum battle therapy), bone setting, spine alignment, qigong, divination, written charms, shaman, talisman, incense ash, or other folk therapy to ease physical discomfort in a nonmedical location (government-certified traditional Chinese medicine and Western medicine excluded)?

Traditional Chinese medicine (TCM) includes herbal medicine, acupuncture, moxibustion, bone reduction, traditional trauma treatment, traditional dislocation treatment, traditional fracture treatment, tui-na, ba-guan, and other therapies. TCM practitioners include registered TCM physicians who are certified, licensed, and practicing in a hospital or clinic setting. TCM in Taiwan is recognized as a health service and treatment modality, and TCM physicians can legally advertise the medical benefits of TCM and claim reimbursement from the National Health Insurance Program [17].

We calculated the density of TCM physicians (TCM physicians/10,000 persons) by using the number of TCM physicians per 10,000 persons for each administrative unit. The first, second, and third tertiles were considered areas of low, moderate, and high TCM physician density, respectively [17, 31].

Regarding unhealthy lifestyle factors, Taiwan has relatively high prevalence of cigarette smoking, alcohol drinking, and betel quid chewing [32]. Tobacco smokers were classified as those who had a few occasions of smoking over their lifetime, those who smoked less than five packs over their lifetime, and those who smoked more than five packages over their lifetime. Our study considered tobacco smokers and current drinkers of any type of alcoholic beverage as well as those who chewed betel quid as having unhealthy lifestyle factors associated with cancer and other diseases [17, 33]. These unhealthy lifestyle factors (alcohol drinking, smoking, and chewing betel quid) were categorized as zero, one, and two or three.

### 2.4. Statistical Analysis

We used chi-square tests to compare differences in sociodemographic factors, lifestyles, and medical utilization between people with and without folk therapy use. Fisher's exact test was used when sample size was small. We used stepwise multivariate logistic regression analysis to estimate the odds ratios (ORs) and corresponding 95% confidence interval (CIs) factors associated with folk therapy use. These covariates included age, gender, family income, ethnicity, marital status, unhealthy lifestyle factors, and density of TCM physicians. For each covariate, we assign a predicted score as risk index according to the significant adjusted OR, and the predicted score is proportional to OR. Utilization predictive score was defined as follows: when 1.0 ≤ OR < 1.5 utilization predicted score is 1, when 1.5 ≤ OR < 2.0 utilization predicted score is 2, when 2.0 ≤ OR < 2.5 utilization predicted score is 3, and when 2.5 ≤ OR < 3.0 utilization predicted score is 4. For investigating the impacts of five major factors (emergency care, hospitalized care, WM outpatient care, frequency of WM outpatient care, and medical expenditure for WM) on the use of folk therapy, we performed five multivariate logistic regression models. Further multivariate logistic regression models were performed to investigate the impacts of use of TCM, number of visits for TCM, use of acupuncture, number of visits for acupuncture treatment, frequency of TCM use, expenditure for TCM, and acupuncture, herbal medicine, and tui-na on the use of folk therapy. Each model adjusted for age, gender, ethnicity, density of TCM physicians, unhealthy lifestyle factors, and medical care in past 6 months in multiple logistic regressions. All analysis was performed using Statistical Analysis Software, version 9.2 (by SAS Institute Inc., Cary, North Carolina, USA). A two-sided *p* value smaller than 0.05 was considered significant.

## 3. Results

The prevalence of utilization of folk therapy within the past one month was 6.8% in 16,750 participants aged 20 years and older (Table 1). A comparison of the demographic characteristics of users versus nonusers of folk therapy revealed that users of folk therapies were significantly older (percentage of using folk therapy of 30–39, 40–49, and 50–59 age groups is 7.94%, 7.63%, and 7.19%, resp., *p* < 0.001). Women had a higher percentage of use of folk therapy than men (7.85% versus 5.74%, *p* < 0.001). Participants who are nonindigenous (6.85% versus 3.93%, *p* = 0.021), who have one or two unhealthy lifestyle factors (7.03% and 6.97% versus 6.55%, *p* < 0.001), who live in areas with moderate or high density of TCM physicians (6.45% and 8.27% versus 5.80%, *p* < 0.001), and who were ill with or without seeking medical care in the previous six months had higher percentages using folk therapy. The two groups were comparable in marital status, employment status, family income, and educational levels. Second, in the multivariate logistic regression analysis, the following groups were more likely to use folk therapy: women (OR = 1.63, 95% CI = 1.40–1.90), ages 30–39, 40–49, and 50–59 (OR = 1.98, 95% CI = 1.49–2.63; OR = 1.63, 95% CI = 1.40–1.90; OR = 1.63, 95% CI = 1.40–1.90, resp.), nonindigenous respondents (OR = 1.90, 95% CI = 1.14–3.17), those with one or two unhealthy lifestyle factors listed above (OR = 1.25, 95% CI = 1.06–1.47 and OR = 1.51, 95% CI = 1.26–1.81, resp.), those who lived in areas where the density of TCM practitioners was moderate or high (OR = 1.08, 95% CI = 0.93–1.26 and OR = 1.40 95% CI = 1.20–1.62, resp.), and those who had been ill with or without medical care in the previous six months (OR = 2.04, 95% CI = 1.73–2.40 and OR = 2.11, 95% CI = 1.76–2.53, resp.).

Table 2 shows the variables of medical care associated with use of folk therapy in the multivariate logistic regression models adjusting for age, gender, ethnicity, density of TCM physicians, unhealthy lifestyle factors, or medical care in past six months. Participants who used Western medicine without prescription (OR = 1.36, 95% CI = 1.18–1.58) and people who received emergency and inpatient or outpatient Western medical care within the previous year (OR = 1.46, 95% CI = 1.24–1.71; OR = 1.28, 95% CI = 1.04–1.57; OR = 1.22, 95% CI = 1.01–1.49, resp.) were more likely to use folk therapy than the comparison group. In addition, the frequency of outpatient Western medical care and expenditure for Western medicine were factors associated with use of folk therapy. People who had higher frequency of or expenditure on outpatient care in the previous year had higher odds ratios of using folk therapy (OR = 1.34, 95% CI = 1.13–1.58 and OR = 1.42, 95% CI = 1.20–1.67, resp.).

Table 3 shows the correlation between TCM use and use of folk therapy in the multivariate logistic regression models adjusting for age, gender, ethnicity, density of TCM physicians, unhealthy lifestyle factors, and medical care in the previous six months. Participants who purchased Chinese herbs without a prescription (OR = 2.26, 95% CI = 1.84–2.78) and people who used TCM and acupuncture in the previous year (OR = 1.93, 95% CI = 1.70–2.19 and OR = 2.59, 95% CI = 2.17–3.09, resp.) were more likely to use folk therapy than the comparison group. In addition, the frequency of TCM use and expenditure on TCM also were associated with use of folk therapy. People who had higher frequency of or expenditure on TCM use in the previous year had higher odds ratios of using folk therapy (OR = 2.87, 95% CI = 2.37–3.47 and OR = 2.57, 95% CI = 2.16–3.04, resp.).

Using the results of Table 1, we calculated the total predictive scores of folk therapy use for every participant. The predictive score for the use of folk therapy was categorized into 0–5, 6-7, 8, and 9-10 scores (Table 4). Compared with people with predictive scores of 0–5, the OR of the use of folk therapy for those with 6-7, 8, and 9-10 scores was 1.97 (95% CI = 1.63–2.39), 2.54 (95% CI = 2.09–3.08), and 2.76 (95% CI = 2.24–3.40), respectively.

## 4. Discussion

While a nationwide survey showed that the prevalence of folk therapy use in the previous month was 6.8%, this study's analysis found that use of acupuncture and frequency of and expenditure on TCM and combined use of acupuncture, tui-na, and herbal medicine were factors associated with use of folk therapy.

The current investigation included 16,750 participants, and on average more than one in fifteen people had used folk therapy within the previous month. The results of this study are similar to those of previous investigations [12, 34] showing 6–8% prevalence in previous month but relatively higher than Tsai's study that found prevalence of 1.3% [25]. This difference may result from different study questionnaires, as Tsai's survey asked: “What is (are) the most common action(s) you take when you experience physical discomfort?” Our study asked: “Have you used folk therapy to deal with physical discomfort in the past month?”

We found a higher prevalence of CAM use among the middle-aged group (30–59 years old) and females, which is similar to previous findings that age and gender are associated with CAM use [31–34]. The results of this study were consistent with previous studies showing that younger people had more knowledge of TCM and more frequently used TCM services to improve their well-being and treat disease symptoms [17, 24]. Middle-aged people are often family caregivers, so they might seek such treatment for their children or parents as well. Gender was also an important factor associated with use of folk therapy. Women may be more willing to improve their health by trying various therapies which are not included in the insurance system. In addition, it is easier for women than men to access folk therapy through social networks [12].

In the present study, we found that people who are nonindigenous and live in areas with a high density of TCM physicians had a higher OR of using folk therapy. Economic growth is a major determinant of physician supply and medical consumption [35]. In general, residents in urban areas have more access to medical services than rural residents, because more physicians practice in cities [13, 33, 34]. Therefore, people living in areas with a high density of TCM physicians have more access to conventional and unconventional therapies [13, 33, 34]. Explanations of why ethnicity differences exist are complex. Because ethnicity interacts with many factors such as culture, nationality, and religion and because ethnicity is closely associated with socioeconomic status differences that affect patterns of accessing care, health status, and other predictors of conventional health services use [36], it is important to determine whether ethnic differences in folk therapy use emerge or persist when several of these predictors are considered simultaneously. In addition, people who had a greater number of unhealthy behaviors were more likely to use folk therapy. Shih et al. [12] suggested that people who have more unhealthy behaviors might consider folk therapy as a form of preventive medicine.

Western medicine is the most popular and conventional treatment in Taiwan and is covered by NHI. Previous studies in Western and Asian countries found that various problems or diseases of human organ systems recognized in Western medicine are also indications for CAM use, including problems of the musculoskeletal, respiratory, and digestive systems, neurological and psychological disorders, and general complaints [16, 21]. According to previous reports, most people consult CAM practitioners for chronic pain resulting from chronic conditions or musculoskeletal system disorders [24]: more than 46.2% of indications for acupuncture visits in Taiwan were for musculoskeletal complaints [37]. These findings might indicate that some disease categories between CAM and Western medicine overlap. We found that people with higher frequencies of using or higher expenditure on Western medicine had a greater tendency to use folk therapy. A possible explanation is that those who had higher use of medical services may care more about their health. Folk therapy can be a second option for people who have more health knowledge or who wish to pursue greater well-being. Clinical physicians need to pay more attention to their patients' use of folk therapy. The importance of predicted scores such as those in the current research is that they can help clinical physicians to identify characteristics of patients who are likely to use folk therapy.

TCM, like Western medicine, has been commonly used by the Taiwanese for problems and diseases of major organ systems. Some TCM treatments are similar to folk therapies. However, practitioners of folk therapy are not licensed and cannot verify that their therapy has any definite medical effects [22]. After adjusting for socioeconomic status, lifestyle choices, and treatment-seeking behaviors in a multivariate logistic regression, we found that many TCM-related variables were associated with use of folk therapy. Because some parts of TCM are similar to folk therapy, it was reasonable that TCM users also used folk therapy, and indeed we found that people with higher frequencies of or expenditure on TCM use had higher OR to utilize folk therapy. In addition, because acupuncture is an invasive treatment, people who use acupuncture may be more likely to try other medical care modalities, such as folk therapy, than those who do not use acupuncture. This study's results reveal that the more people use acupuncture, the higher the probability that these same people will use folk therapy. Finally, the therapeutic methods of TCM involve different approaches, such as acupuncture, tui-na bodywork, and herbal medicine. We found that people who used more of the therapeutic methods mentioned above were more likely to try folk therapy as another wellness-seeking behavior. In short, similar health issues might motivate people in Taiwan to seek help from licensed Western medicine and TCM physicians as well as consulting noncertified folk therapists.

This study has some limitations. First, we could not provide information about whether folk therapy use is increasing or decreasing over time, because our research is based on a cross-sectional population-based survey. Second, the causality between folk therapy and associated factors also could not be confirmed in this cross-sectional study. Third, some information used in this study was based on self-reported questionnaire results, so recall bias may be a factor. Fourth, because use of folk therapy was a dichotomous dependent variable, information on the number of folk therapies used and frequency of their use was lost. It may be that substantial differences exist between heavy and light users of one or more CAM modalities [17].

In conclusion, this study reported that the prevalence of folk therapy use among adults in Taiwan within a certain month was 6.8%, suggesting that people frequently use both folk therapy and conventional medicine in the same period when they feel unwell. Use of folk therapy was associated with age, gender, ethnicity, lifestyle choices, and use of Western medical services. We suggest that physicians need to understand their patients' use of folk therapy so that their Western medical treatments might have the best chance of effectiveness [18, 20] and to avoid possible adverse effects from folk therapy interactions with Western medications. Health authorities also need to establish appropriate regulations for folk therapy to protect patients' health.

## Figures and Tables

**Table 1:** Characteristics of users and nonusers of folk therapy.

	Use of folk therapy						
	No (*N* = 15615)		Yes (*N* = 1135)		OR	(95% CI)^‡^	Scores^§^
	*n*	(%)	*n*	(%)			
Age (years)							
20–29	3399	(93.3)	243	(6.7)	1.66	(1.25–2.22)	2
30–39	3198	(92.1)	276	(7.9)	1.98	(1.49–2.63)	2
40–49	3416	(92.4)	282	(7.6)	1.92	(1.45–2.55)	2
50–59	2387	(92.8)	185	(7.2)	1.83	(1.37–2.46)	2
60–69	1600	(95.1)	82	(4.9)	1.22	(0.87–1.71)	1
≥70	1615	(96.0)	67	(4.0)	1.00	(Reference)	0
Gender							
Male	8057	(94.3)	491	(5.7)	1.00	(Reference)	0
Female	7558	(92.2)	644	(7.8)	1.63	(1.40–1.90)	2
Occupation							
White-collar	5626	(92.5)	459	(7.5)	—	—	—
Blue-collar	5775	(93.9)	377	(6.1)	—	—	—
Others	4241	(93.4)	299	(6.6)	—	—	—
Education (years)							
0	1188	(95.2)	60	(4.8)	—	—	—
1–9	5389	(93.7)	361	(6.3)	—	—	—
10–12	4406	(92.8)	342	(7.2)	—	—	—
≥13	4632	(92.6)	372	(7.4)	—	—	—
Family income (USD/month)							
<1000	4134	(94.1)	258	(5.9)	—	—	—
1000–6667	11176	(93.0)	836	(7.0)	—	—	—
>6667	405	(90.8)	41	(9.2)	—	—	—
Marital status							
Married	10003	(93.2)	734	(6.8)	—	—	—
Unmarried	3781	(93.0)	284	(7.0)	—	—	—
Others	1831	(94.0)	117	(6.0)	—	—	—
Ethnicity							
Nonindigenous	15224	(93.2)	1119	(6.8)	1.90	(1.14–3.17)	2
Indigenous	391	(96.1)	16	(3.9)	1.00	(Reference)	0
Density of TCM physicians^*∗*^							
Low	5649	(94.2)	348	(5.8)	1.00	(Reference)	0
Moderate	5252	(93.6)	362	(6.4)	1.08	(0.93–1.26)	1
High	4714	(91.7)	425	(8.3)	1.40	(1.20–1.62)	1
Unhealthy lifestyle factors^†^							
None	7722	(93.5)	541	(6.5)	1.00	(Reference)	0
One	3491	(93.0)	264	(7.0)	1.25	(1.06–1.47)	1
Two or three	4402	(93.0)	330	(7.0)	1.51	(1.26–1.81)	2
Medical care in past 6 months							
Without illness	4945	(96.0)	203	(4.0)	1.00	(Reference)	0
Illness with care	7252	(92.3)	606	(7.7)	2.04	(1.73–2.40)	3
Illness without care	3438	(91.3)	326	(8.7)	2.11	(1.76–2.53)	3

^*∗*^TCM: traditional Chinese medicine.

^†^Including alcohol drinking, smoking, and betel nut chewing.

^‡^Using stepwise selection and the variables in the multiple logistic regression model included age, gender, ethnicity, density of TCM physicians, unhealthy lifestyle factors, and medical care in past 6 months. Occupation, family income, and marital status were not included in the final model because they were not significant factors.

^§^Predicted scores: 1.0 ≤ OR < 1.5 predicted score = 1, 1.5 ≤ OR < 2.0 predicted score = 2, 2.0 ≤ OR < 2.5 predicted score = 3, 2.5 ≤ OR < 3.0 predicted score = 4, 3.0 ≤ OR < 3.5 predicted score = 5, and 3.5 ≤ OR < 4.0 predicted score = 6.

**Table 2:** Use of Western medicine within previous year in association with use of folk therapy in most recent month.

	Use of folk therapy					
Medical utilization within previous year	No (*N* = 15615)		Yes (*N* = 1135)		OR	(95% CI)^*∗*^
	*n*	(%)	*n*	(%)		
Emergency care (Model 1)						
No	13519	(93.6)	920	(6.4)	1.00	(Reference)
Yes	2096	(90.7)	215	(9.3)	1.46	(1.24–1.71)
Hospitalized care (Model 2)						
No	14383	(93.4)	1023	(6.6)	1.00	(Reference)
Yes	1232	(91.7)	112	(8.3)	1.28	(1.04–1.57)
WM outpatient care (Model 3)						
No	4833	(93.9)	313	(6.1)	1.00	(Reference)
Yes	10782	(92.9)	822	(7.1)	1.22	(1.01–1.49)
Frequency of WM outpatient care (Model 4)						
None	4839	(93.9)	313	(6.1)	1.00	(Reference)
Low	3650	(94.0)	235	(6.0)	0.93	(0.78–1.12)
Moderate	3570	(92.8)	278	(7.2)	1.05	(0.89–1.25)
High	3556	(92.0)	309	(8.0)	1.34	(1.13–1.58)
Medical expenditure for WM (Model 5)						
None	4839	(93.9)	313	(6.1)	1.00	(Reference)
Low	3601	(94.1)	226	(5.9)	0.89	(0.74–1.07)
Moderate	3556	(92.9)	271	(7.1)	1.02	(0.86–1.21)
High	3619	(91.8)	325	(8.2)	1.42	(1.20–1.67)

WM: Western medicine.

^*∗*^There were 5 multivariate logistic regression models performed to investigate the impacts of five major factors (emergency care, hospitalized care, WM outpatient care, frequency of WM outpatient care, and medical expenditure for WM) on the use of folk therapy. Each model adjusted for age, gender, ethnicity, density of TCM physicians, unhealthy lifestyle factors, and medical care in past 6 months in multiple logistic regressions.

**Table 3:** Use of traditional Chinese medicine within previous year in association with use of folk therapy in most recent month.

	Use of folk therapy					
	No (*N* = 15615)		Yes (*N* = 1135)		OR	(95% CI)^*∗*^
	*n*	(%)	*n*	(%)		
Use of TCM (Model 1)^*∗*^						
No	11879	(94.6)	679	(5.4)	1.00	(Reference)
Yes	3736	(89.1)	456	(10.9)	1.93	(1.70–2.19)
Number of visits for TCM (Model 2)^*∗*^						
None	11879	(94.6)	679	(5.4)	1.00	(Reference)
1 visit	1164	(92.0)	101	(8.0)	1.42	(1.14–1.77)
2 visits	649	(91.3)	62	(8.7)	1.52	(1.16–2.01)
≥3 visits	1923	(86.8)	293	(13.2)	2.36	(2.04–2.74)
Use of acupuncture (Model 3)^*∗*^						
No	14684	(93.8)	964	(6.2)	1.00	(Reference)
Yes	931	(84.5)	171	(15.5)	2.59	(2.17–3.09)
Number of visits for acupuncture treatment (Model 4)^*∗*^						
Without TCM use	11879	(94.6)	679	(5.4)	1.00	(Reference)
Use of TCM but without acupuncture	2805	(90.8)	285	(9.2)	1.60	(1.38–1.85)
1 visit	297	(90.3)	32	(9.7)	1.65	(1.13–2.40)
2 visits	285	(86.1)	46	(13.9)	2.59	(1.87–3.59)
≥3 visits	349	(79.0)	93	(21.0)	4.35	(3.40–5.56)
Frequency of TCM use (Model 5)^*∗*^						
None	11879	(94.6)	679	(5.4)	1.00	(Reference)
Low	1813	(91.8)	163	(8.2)	1.46	(1.22–1.75)
Moderate	1064	(88.9)	133	(11.1)	1.95	(1.60–2.38)
High	859	(84.3)	160	(15.7)	2.87	(2.37–3.47)
Expenditure for TCM (Model 6)^*∗*^						
None	11881	(94.6)	680	(5.4)	1.00	(Reference)
Low	1235	(91.2)	119	(8.8)	1.57	(1.28–1.93)
Moderate	1274	(90.5)	134	(9.5)	1.64	(1.35–2.00)
High	1225	(85.8)	202	(14.2)	2.57	(2.16–3.04)
Acupuncture, herbal medicine, and tui-na (Model 7)^*∗*^						
None (including other TCM)	11881	(94.6)	679	(5.4)	1.00	(Reference)
Acupuncture only	176	(88.4)	23	(11.6)	2.17	(1.38–3.42)
Herbal medicine only	1841	(91.6)	168	(8.4)	0.10	(0.06–0.17)
Tui-na only	367	(90.8)	37	(9.2)	1.31	(0.92–1.85)
Acupuncture and herbal medicine	370	(84.9)	66	(15.1)	2.23	(1.70–2.94)
Acupuncture and tui-na	97	(92.4)	8	(7.6)	1.12	(0.54–2.32)
Herbal medicine and tui-na	595	(88.2)	80	(11.8)	1.68	(1.31–2.15)
All	288	(79.6)	74	(20.4)	3.30	(2.52–4.31)

CI: confidence interval; OR: odds ratio; TCM: traditional Chinese medicine.

^*∗*^There were seven multivariate logistic regression models performed to investigate the impacts of seven major factors (use of TCM, number of visits for TCM, use of acupuncture, number of visits for acupuncture treatment, frequency of TCM use, expenditure for TCM, and acupuncture, herbal medicine, and tui-na) on the use of folk therapy. Each model adjusted for age, gender, ethnicity, density of TCM physicians, unhealthy lifestyle factors, and medical care in past 6 months in multiple logistic regressions.

**Table 4:** Predictive scores for use of folk therapy among adults in Taiwan.

	Use of folk therapy			
	Interviewees	Users	OR	(95% CI)
Predictive scores^*∗*^				
0–5	4365	156	1.00	(Reference)
6-7	5789	395	1.97	(1.63–2.39)
8	4086	351	2.54	(2.09–3.08)
9-10	2510	233	2.76	(2.24–3.40)

CI: confidence interval; OR: odds ratio.

^*∗*^Using the results of Table 1, we calculated the total predictive scores of folk therapy use for every participant.
