# Erratum to “Use of Folk Therapy in Taiwan: A Nationwide Cross-Sectional Survey of Prevalence and Associated Factors”

**DOI:** 10.1155/2019/8498416

**Published:** 2019-02-05

**Authors:** Evidence-Based Complementary and Alternative Medicine

In the article titled “Use of Folk Therapy in Taiwan: A Nationwide Cross-Sectional Survey of Prevalence and Associated Factors” [1], an administrative error meant the editor originally named on the article did not handle the review process. The article and its peer review have been reassessed by Morry Silberstein, who found no concerns.
